# Isolation with differentiation followed by expansion with admixture in the tunicate *Pyura chilensis*

**DOI:** 10.1186/1471-2148-13-252

**Published:** 2013-11-15

**Authors:** Pilar A Haye, Natalia C Muñoz-Herrera

**Affiliations:** 1 Laboratorio de Diversidad Molecular, Departamento de Biología Marina, Facultad de Ciencias del Mar, Universidad Católica del Norte, Casilla 117, Coquimbo, Chile; 2 Centro de Estudios Avanzados en Zonas Áridas, Coquimbo, Chile; 3 Interdisciplinary Center for Aquaculture Research (INCAR), Universidad de Concepción, Casilla 160-C, Concepción, Chile

**Keywords:** Phylogeography, Genetic structure, Dispersal potential, Short-lived larvae, Connectivity, Biofouling, COI, Elongation Factor 1 alpha

## Abstract

**Background:**

*Pyura chilensis*, a tunicate commercially exploited as food resource in Chile, is subject to management strategies, including restocking. The goal of this study was to examine the genetic structure of *P. chilensis* using information from a mitochondrial gene (Cytochrome Oxidase I, COI) and a nuclear gene (Elongation 1 alpha, EF1a)*,* to characterize the geographic distribution of genetic diversity and differentiation, and to identify the main processes that have shaped it. We analyzed 268 and 208 sequences of COI and EF1a, respectively, from samples of eight local populations covering ca. 1800 km.

**Results:**

For *Pyura chilensis*, partial sequences of the gene COI revealed three highly supported haplogroups that diverged 260000 to 470000 years ago. Two haplogroups currently are widely distributed and sympatric, while one is dominant only in Los Molinos (LM, 39°50′S). The two widespread COI haplogroups underwent a geographic expansion during an interglacial period of the Late Pleistocene ca. 100000 years ago. The nuclear gene was less divergent and did not resolve the COI haplogroups. Bayesian clustering of the nuclear gene’s SNPs revealed that individuals from the two widespread COI haplogroups were mostly assigned to two of the three detected clusters and had a marked degree of admixture. The third cluster predominated in LM and showed low admixture. Haplotypic diversity of both genes was very high, there was no isolation by distance, and most localities were genetically undifferentiated; only LM was consistently differentiated with both genes analyzed.

**Conclusions:**

*Pyura chilensis* has less genetic structure than expected given its life history, which could be a consequence of dispersal on ship hulls. The only differentiated local population analyzed was LM. Coincidentally, it is the one furthest away from main maritime routes along the coast of Chile.

The use of mitochondrial and nuclear markers allowed detection of divergent mitochondrial haplogroups in *P. chilensis*, two of which revealed nuclear admixture. The genetic structure of *P. chilensis* has likely been shaped by Pleistocene’s climatic effect on sea level leading to population contraction with isolation, followed by geographic range expansions with concomitant secondary contact and admixture.

## Background

The tunicate *Pyura chilensis* Molina 1782, is a solitary ascidian found along the Humboldt Current System (HCS) from 10°S to 44°S along the southeast Pacific coast [1]. They are found in subtidal rocky habitats, as solitary individuals and as patches that vary in size from a few to thousands of individuals. When living together, they form a matrix structure. The tunics are cemented to the substratum, either attached to rocks or to floating structures such as ropes, buoys, boats and ship hulls. Adult hermaphrodites and a short-lived free larva characterize the life history of *P. chilensis*. It is a digonic protandrous hermaphrodite that even though it has the ability to self-fertilize, cross-fertilization is the main reproductive strategy when conspecifics are close enough [2]. The larvae spend from 12 to 24 hours in the water column, providing the species with a relatively low intrinsic dispersal potential. *Pyura chilensis* is intensively harvested along its distributional range for human consumption, especially towards the southern area. It is considered an important benthic resource and is subject to regulations and management practices. In Chile, restocking programs can be established for benthic resources such as *P. chilensis*, upon request of the fisherman’s association in charge of a management area. *Pyura chilensis* is not only relevant as a resource by itself, but also as an ecosystem component that favors the presence of the highly priced muricid gastropod *Concholepas concholepas*. Because of its ecological and economic relevance, restocking programs have been undertaken even in the absence of baseline knowledge of the genetic structure of the species. The only population genetic study to date on *P. chilensis* was carried out using two allozyme loci and three local populations [3]. The study revealed slight but significant differentiation between individuals from the southernmost locality analyzed. Given the low number of loci and populations analyzed, the result can be considered as preliminary with respect to the understanding of the genetic structure of the species. However, the greater differentiation of the southern populations could be reflecting a real signature of the geographic variation of the genetic diversity of *P. chilensis*. Since allozymes are highly conserved markers when compared to DNA sequences, individuals of *P. chilensis* from the southern area of the species geographic distribution will probably also be significantly differentiated with commonly used phylogeographic markers such as mitochondrial DNA.

Phylogeographic patterns of marine species do not follow a simple predictive arrangement within a biogeographic region or a taxon and contradictory results have highlighted the overall complex idiosyncrasy of genetic structure patterns (e.g., [4-9]). Exceptionally, the geographic structure of the genetic diversity of coastal species along the HCS has shown a consistent association between dispersal potential (either as larvae or alternative means) and the degree of genetic homogeneity based on several analyses of mitochondrial DNA sequence data (e.g. [5,10-16]). Additionally, most taxa with low dispersal potential studied so far along the HCS display a phylogeographic break coincident with a biogeographic transition zone in the vicinity of 30-33°S in the Chilean coast (e.g. [4,11,14-16]).

Although scarcely studied along the coast of Chile, paleoclimatic events such as the Pleistocene climatic oscillations have been shown to shape the genetic structure of some marine taxa. The last 130000 years have been characterized by strong climatic variations in sea-surface temperature and sea level. Species with short-lived larvae may have been more affected by sea-level changes, because local populations may become isolated during low sea level due to vicariant barriers. Additionally, species with broad geographic distributions with respect to their dispersal potential and species with large population sizes are more likely to show past evolutionary events (e.g. [17]). Subsequent sea level rises could provide the opportunity for secondary contact between divergent lineages [17,18]. Depending on interbreeding capabilities, secondary contact could lead to greater variability within the species or cryptic speciation. The Pleistocene contraction-expansion model invokes repeated population fluctuations linked to glacial and interglacial periods [9,19] and that have engendered genetic signatures on several marine species [19-22]. Even in the same geographic area species may differ in the effect of past paleoclimatic events, and on which event triggered the effect. For example, Pleistocene climatic oscillations, including the last glacial maximum (LGM) and previous events, have affected some coastal species of the northeastern Pacific while others show stasis [9]. For the coast of Chile, paleoclimate’s influence has been suggested as a possible cause for transient allopatry, resulting in two closely related lineages of the kelp *Lessonia*[11]. Other phylogeographic studies that focused on southern Chile, at 43°S and higher latitudes where Pleistocene glaciation cycles were intense, also have linked genetic patterns with paleoclimate [5,16]. However, for taxa that live along the HCS, there are no clear examples of the effects of paleoclimate on current genetic structure.

The goal of this study was to characterize the genetic structure of *P. chilensis* along ca. 1800 km with data from the mitochondrial gene Cytochrome Oxidase I (COI), and the nuclear gene Elongation Factor 1 alpha (EF1a), to determine the geographic distribution of the genetic diversity and differentiation, and evaluate the main drivers of the genetic structure. Species of the HCS with low dispersal potential (either larval or alternative means) are expected to have strong genetic structure, a phylogeographic break at 30°S, and a pattern of isolation by distance. Results will provide a base-line scenario of the genetic diversity of *P. chilensis* to orient decision-making associated with management strategies, specifically restocking practices.

Results revealed a contraction-expansion scenario of *Pyura chilensis* COI lineages associated to Pleistocene’s climatic oscillations, specifically sea level. COI lineages with wide sympatric geographic distribution had a degree of nuclear admixture consistent with lack of reproductive isolation of COI lineages. Only one locality had individuals highly differentiated, with little nuclear admixture, and population substructure, but overall, *P. chilensis* showed a greater degree of homogeneity than expected given its life history.

## Results

### Phylogenetics of *Pyura chilensis*

Partial sequences of the COI and EF1a genes were obtained from individuals of *Pyura chilensis* from eight local populations along the coast of Chile from 26°08′S to 41°52′S (Table 1). Final truncated alignments consisted of 268 and 208 sequences of 614 and 319 nucleotides in length, for COI and EF1a, respectively. Alignments of both genes had full open reading frames and matched with coding regions of the respective genes from GenBank. The gene COI had a larger number of variable sites (150) than EF1a (with 12). Of the variable sites, 102 (COI) and 7 (EF1a) were parsimony informative. Data sets did not have evidence of mutation saturation (*P* = 0) considering all data together or for each codon position separately. Neutrality tests based on rates of synonymous (dS) and non-synonymous (dN) substitutions, did not allow rejecting the null hypothesis of neutral evolution for COI and EF1a sequences. Both codon-based and sequence analyses dN were significantly lower than dS (*P* > 0.05).

**Table 1:** ***Pyura chilensis, *** **sampling localities and number of individuals analyzed of COI and EF1a**

**Site name**	**Coordinates**	**Acronym**	**COI N**	**EF1a N**
Pan de Azúcar	26°08′S-70°39′W	PA	30	28
Caleta Pajonales	27°44′S-71°02′W	CP	31	27
Punta de Choros	29°14′S-71°28′W	PC	33	23
La Herradura	29°58′S-71°21′W	LH	37	29
Punta Lengua de Vaca	30°15′S-71°38′W	PV	32	25
Talcahuano	36°42′S-73°06′W	TL	27	18
Los Molinos	39°50′S-73°23′W	LM	38	31
Ancud	41°52′S-73°49′W	AC	40	27
TOTAL			268	208

Cytochrome Oxidase I (COI); Elongation Factor 1 alpha (EF1a).

**Figure 1:**
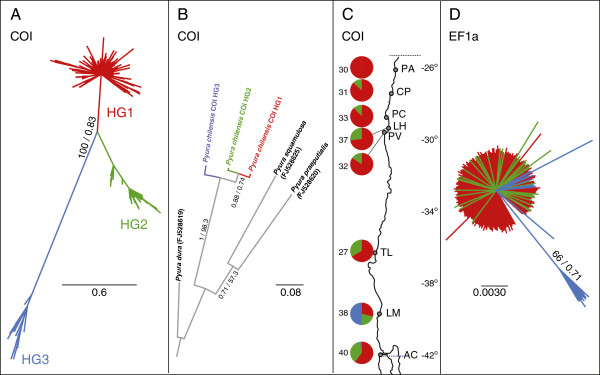
**Mitochondrial COI haplogroups and sequences of EF1a of *Pyura chilensis. *A)** Bayesian unrooted phylogram of COI sequences of *P. chilensis*; node support corresponds to bootstrap values greater than 50 from the maximum likelihood analysis, and to Bayesian posterior probabilities greater than 0.7; **B)** Bayesian phylogenetic tree of COI haplogroups rooted with three outgroup taxa of the genus *Pyura*; node support as in **A**. **C)** Geographic distribution of COI haplotypes. Each pie represents 100% of the sampled haplotypes, and the subdivisions represent relative frequencies of each haplogroup per locality; **D)** Bayesian unrooted phylogram of un-Phased EF1a sequences; support for nodes as in **A**. **A**-**D**) In all figures colors represent COI haplogroups; red for COI HG1, green for COI HG2 and blue for COI HG3.

Phylogenetic reconstructions of the gene COI revealed three highly divergent, reciprocally monophyletic, and well-supported lineages within *P. chilensis* (Figure 1a). Hereon these COI haplogroups will be referred to as HG1, HG2 and HG3 according to their relative abundances among the analyzed individuals of *P. chilensis*. The three haplogroups of *P. chilensis* showed a considerably lower divergence among them than there was between species pairs of the genus *Pyura* (Figure 1b). The COI HG3 was the basal haplogroup of *P. chilensis* (Figure 1b); this was also the most differentiated haplogroup bearing the longest branch (Figure 1a, 1b). Estimated times of divergence of the COI data based on a 3% Myr^-1^ mutation rate indicated that from an ancestral lineage of *P. chilensis*, HG2 and HG3 diverged ca. 470000 years ago, while HG1 diverged from HG2 260000 years ago. These time estimates have to be interpreted cautiously because of uncertainties in mutation rate, which could be much greater than the already high 3% Myr^-1^ rate considered [23-26]. Estimated dates matched cold Marine Isotope Stages (MIS) 8 and 12 of the Pleistocene, cool glacial periods of low sea level.

The geographic distribution of the COI haplogroups reveals that two of them are sympatric throughout most of the range analyzed. The southern area has a greater overall COI diversity, because all three haplogroups are present there (Figure 1c). COI HG1 was found at all sites, with a preponderant presence, and was the only one present in the northernmost site PA (26°08′S). This was the most abundant haplogroup at all localities excepting LM (39°50′S) (Figure 1c). HG2 was found at CP (27°70′S) and southward (only not present in PA), with greater frequency in the southern area, specifically in TL (36°42′S) and AC (41°52′S) (Figure 1c). Lastly, HG3 had a geographic distribution restricted to the two southernmost localities (LM and AC), and had greater relative abundance in LM (only one individual in AC) (Figure 1c).

Most EF1a sequences grouped in a star-like relationship, which separated with moderate support from a clade composed of 52.6% of the individuals of the HG3 of LM (Figure 1d). The EF1a gene tree neither provided resolution for COI HG1 or HG2, nor for almost half of the individuals of the HG3.

### Genetic structure of the EF1a SNPs of *Pyura chilensis*

The EF1a sequence data had eight polymorphic sites (SNPs) with over 5% of polymorphism. These were used to perform diallelic analyses, albeit using caution, given the highly linked loci analyzed. Locus by locus and site by site tests for Hardy-Weinberg equilibrium (HWE) denoted 9 out of 72 comparisons out of equilibrium (Additional file 1: Table S1), six of them were in LM. Most of these departures were associated with positive and significant *F*_*IS*_ values (Additional file 1: Table S1 and S2). Likewise, multilocus probabilities associated to HWE were non-significant except for LM (*P* = 0) (Table 2). When LM is subdivided according to the two divergent groups (Figure 1d), both groups of LM accommodate to Hardy-Weinberg expectations, suggesting that the lack of HWE in LM is due to a Wahlund effect.

**Table 2:** **Summary statistics for the nine EF1a SNPs of *Pyura chilensis***

**Site**	**He**	**Ho**	***P *** **(HWE)**	***F***_***IS***_	***F***_***ST***_
PA	0.219	0.238	0.846	-0.089	0.038
CP	0.272	0.280	0.975	-0.029	0.041
PC	0.262	0.246	0.848	0.060	0.027
LH	0.229	0.176	0.160	**0.233**	0.028
PV	0.236	0.240	0.950	-0.019	0.028
TL	0.290	0.235	0.691	**0.195**	0.035
LM	0.298	0.158	**0.006**	**0.476**	**0.141**
AC	0.279	0.292	0.983	-0.050	0.026
TOTAL	0.260	0.233	0.682	0.144	0.055

Expected heterozygosity (He); Observed heterozygosity (Ho); Probability of deviation from Hardy-Weinberg equilibrium (*P* (HWE)); *P* (HWE) in bold indicate significant HWE departures after Bonferroni correction; inbreeding coefficient (*F*_*IS*_); fixation coefficient (*F*_*ST*_); significant values are in bold (*P* < 0.05).

**Figure 2:**
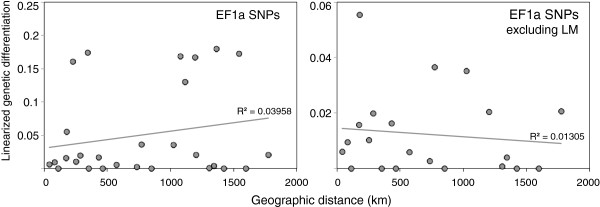
**Relationship between genetic differentiation of EF1a SNPs and geographic distance in *Pyura chilensis*.** The relationship was explored on all the EF1a SNP data (left) and excluding the locality LM (right).

**Figure 3:**
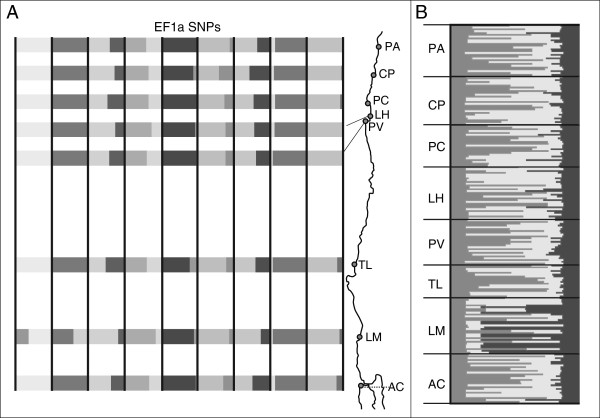
**Genetic structure of EF1a SNPs of *Pyura chilensis. *A)** Relative frequencies per locality of 9 polymorphic SNPs, each “column” represents a locus and the bands of different grey shades correspond to the relative frequency of alleles; **B)** Genetic composition of individuals inferred from Bayesian clustering analysis. Best fit of data with k = 3 (Additional file 1: Figure S1).

Based on the EF1a SNPs, only LM was significantly differentiated at the site level (Table 2). Population pairwise *F*_*ST*_ values were in general low and non-significant; only LM was significantly differentiated from all other populations (Additional file 1: Table S3). Isolation by distance was also non-significant (Mantel’s correlation coefficient’s *P* > 0.05) (Figure 2), and allele frequencies were similar between localities; only LM showed marked differences (Figure 3a).

Bayesian clustering analysis also supports the distinctiveness of LM based on EF1a SNPs. Three clusters were recovered in the most likely scenario (Additional file 1: Figure S1); two widespread and currently admixed, and one that was present mostly in LM and that showed very low admixture (Figure 3b). Over 50% of the individuals of COI HG1 and COI HG2 were assigned with greater probability to Bayesian K-cluster 2. For these two haplogroups, there was also a high percentage of individuals assigned with greater probability to k-cluster 3 (36.77% and 44.12%, for HG1 and HG2 respectively), showing that these two haplogroups have mixed nuclear ancestry. Very few individuals of these two COI haplogroups were assigned to k-cluster 1, 4.51% and 0% for HG1 and HG2, respectively. COI HG3 shows a contrasting pattern, with 84.21% of individuals assigned with greater probability to k-cluster 1, and a smaller percentage assigned to k-clusters 2 and 3, 10.53% and 5.26%, respectively (Additional file 1: Figure S2).

### Genetic structure of haplotype data of COI and EF1a of *Pyura chilensis*

Haplotypic data of the COI gene had 171 haplotypes and the EF1a sequences led to 32 haplotypes. Overall, diversity was very high in both genes analyzed (Table 3). Total haplotypic and nucleotidic diversities were 0.981 and 0.0214 for the COI gene, and 0.907 and 0.0084 for the EF1a gene. All COI haplogroups had the haplotypic diversity over 0.874, while nucleotidic diversity ranged from 0.0046 in HG2 to 0.0093 in HG3 (Table 3).

**Table 3:** **Genetic diversity of COI haplogroups and EF1a haplotypes of *Pyura chilensis***

**Hg**	**Site**	***n***	***H***	***S***	***h***	**π**
**ALL COI**	PA	30	29	38	0.998	0.0069
	CP	31	27	52	0.989	0.0122
	PC	33	26	58	0.983	0.0117
	LH	37	33	60	0.992	0.0163
	PV	32	27	53	0.986	0.0130
	TL	27	16	37	0.920	0.0152
	LM	38	32	78	0.986	0.0401
	AC	40	18	58	0.850	0.0178
	**Total value**	268	171	150	0.981	0.0214
**COI HG1**	PA	30	29	38	0.998	0.0069
	CP	27	24	39	0.989	0.0072
	PC	29	22	43	0.978	0.0070
	LH	27	24	44	0.989	0.0082
	PV	27	24	40	0.989	0.0075
	TL	18	10	18	0.843	0.0039
	LM	11	8	19	0.927	0.0067
	AC	24	8	9	0.612	0.0025
	**Total value**	193	120	111	0.974	0.0066
**COI HG2**	CP	4	3	6	0.833	0.0049
	PC	4	4	7	1.000	0.0062
	LH	10	9	14	0.978	0.0051
	PV	5	3	5	0.700	0.0033
	TL	9	6	10	0.889	0.0054
	LM	8	5	5	0.786	0.0026
	AC	15	9	12	0.905	0.0050
	**Total value**	55	31	32	0.874	0.0046
**COI HG3**	LM	19	19	32	1.000	0.0092
	AC	1	-	-	-	-
	**Total value**	20	20	32	1.000	0.0093
**ALL EF1a**	PA	19	12	8	0.885	0.0073
	CP	18	14	10	0.895	0.0080
	PC	16	12	11	0.893	0.0087
	LH	26	15	10	0.905	0.0070
	PV	21	13	10	0.852	0.0073
	TL	15	15	11	0.940	0.0094
	LM	25	17	12	0.844	0.0081
	AC	22	17	12	0.908	0.0081
	**Total value**	162	32	18	0.907	0.0084

Number of analyzed sequences (*n*); number of haplotypes (*H*); number of segregating sites (*S*); haplotype diversity (*h*); nucleotide diversity (π).

The geographic distribution of the genetic diversity varied between markers and COI haplogroups (Additional file 1: Figure S3). COI HG1 had the highest diversity and number of private haplotypes in northern localities, while towards the south the proportion of shared alleles was greater. For COI HG2 and COI HG3 there is no clear diversity trend; HG2 had more private haplotypes than shared at most sites and HG3 was highly variable with most individuals sampled bearing a different haplotype. EF1a has a slightly greater diversity in the south than the north (Additional file 1: Figure S3).

**Figure 4:**
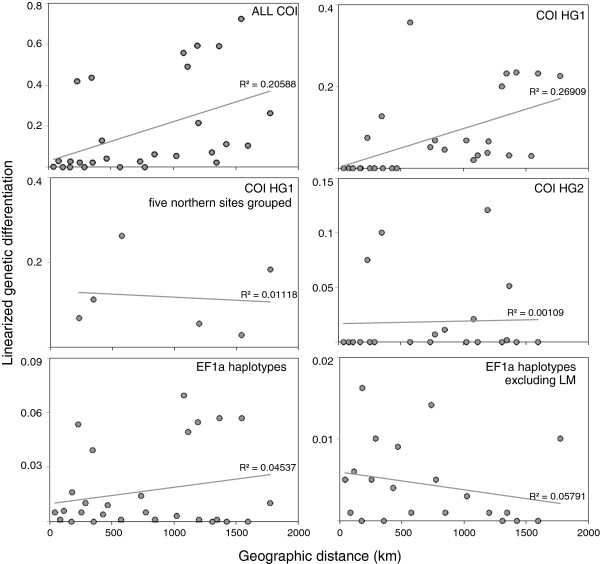
**Genetic differentiation against geographic distance for COI haplogroups and EF1a haplotypes of *Pyura chilensis.*** Linearized genetic differentiation and geographic distance for the following data: ALL COI, COI HG1, CO HG1 grouping the five northern sites, COI HG2, all EF1a, and EF1a excluding LM.

Pairwise genetic differentiation values (Φ_**ST**_ and Snn) of the haplotype data were in general low and non-significant. Just as with the EF1a SNP data, LM was significantly differentiated for the entire COI dataset, COI HG1 and for EF1a haplotypes (Table 4). For the entire COI dataset and for COI HG1, other sites were also significantly differentiated, yet not for COI HG2 or the EF1a haplotype data. COI HG2 appeared undifferentiated at all sites. SAMOVA analyses of haplotypic data consistently separated LM from other localities regardless of the data set used, although the optimal number of groups was generally 3 and the third group varied between data sets (Additional file 1: Table S4). The evaluation of isolation by distance revealed that none of the data sets (or subsets) had significant correlations between genetic differentiation and geographic distance (Mantel’s correlation coefficient’s, *P* > 0.05) and the association between the linearized genetic differentiation and geographic distance showed a poor correlation (Figure 4).

**Table 4:** **Genetic differentiation (Φ**_**ST **_**and Snn) of mitochondrial and nuclear haplotypes of *Pyura chilensis***

		**PA**	**CP**	**PC**	**LH**	**PV**	**TL**	**LM**	**AC**
**ALL COI**	**PA**		0.521	0.532	0.581	0.509	**0.632**	**0.837**	**0.801**
	**CP**	0.026		0.462	0.469	0.413	**0.612**	**0.799**	**0.752**
	**PC**	0.020	-0.176		0.459	0.427	**0.609**	**0.792**	**0.758**
	**LH**	**0.115**	0.020	0.027		0.429	0.567	**0.745**	**0.665**
	**PV**	**0.039**	-0.014	-0.012	0.002		0.549	**0.758**	**0.701**
	**TL**	**0.178**	0.052	0.059	-0.010	0.026		**0.772**	**0.740**
	**LM**	**0.420**	**0.371**	**0.372**	**0.329**	**0.358**	**0.304**		**0.661**
	**AC**	**0.209**	**0.094**	**0.101**	0.020	**0.068**	-0.002	**0.295**	
**COI HG1**	**PA**		0.488	0.501	0.507	0.466	0.564	**0.729**	**0.743**
	**CP**	-0.006		0.487	0.493	0.426	**0.632**	**0.778**	**0.783**
	**PC**	-0.000	-0.005		0.470	0.438	**0.612**	**0.780**	**0.816**
	**LH**	-0.004	-0.007	-0.006		0.449	**0.595**	**0.754**	**0.754**
	**PV**	-0.003	-0.007	-0.005	-0.004		**0.584**	**0.721**	**0.747**
	**TL**	**0.061**	**0.063**	**0.043**	**0.063**	**0.049**		**0.741**	**0.863**
	**LM**	**0.029**	**0.030**	**0.036**	**0.030**	0.020	**0.113**		0.605
	**AC**	**0.184**	**0.188**	**0.190**	**0.188**	**0.167**	**0.263**	0.069	
**COI HG2**	**CP**			0.292	0.374	0.333	0.541	0.547	0.663
	**PC**		-0.051		0.417	0.361	0.596	0.503	0.637
	**LH**		-0.094	-0.004		0.360	0.502	0.457	0.509
	**PV**		-0.126	-0.005	-0.084		0.433	0.514	0.576
	**TL**		-0.083	0.011	0.007	-0.101		0.568	0.526
	**LM**		0.049	0.108	-0.002	0.021	0.091		0.567
	**AC**		-0.048	-0.019	0.002	-0.085	-0.042	0.070	
**COI HG3**	**LM**								
	**AC**							0.065	
**All EF1a**	**PA**		0.520	0.491	0.489	0.479	0.529	0.599	0.511
	**CP**	0.016		0.594	0.509	0.492	0.534	**0.632**	0.462
	**PC**	-0.005	-0.001		0.521	0.473	0.520	**0.628**	0.485
	**LH**	0.004	0.005	0.001		0.482	0.555	**0.589**	0.481
	**PV**	0.009	0.010	0.006	0.005		0.521	**0.603**	0.451
	**TL**	0.001	0.003	0.001	0.005	0.014		0.563	0.484
	**LM**	**0.054**	**0.054**	**0.052**	**0.047**	**0.065**	**0.038**		**0.602**
	**AC**	0.010	-0.006	-0.001	0.001	-0.005	0.001	**0.051**	

Pairwise Φ_ST_ estimates below the diagonal and Snn values above diagonal. Significant values in bold (*P* < 0.05).

**Figure 5:**
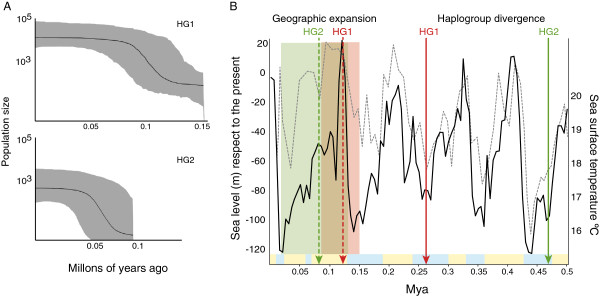
**Bayesian skyline plots and timeline with the estimated divergence and expansion times of two mitochondrial haplogroups of *Pyura chilensis*. A)** Bayesian skyline plots representing historic changes in effective population size of mitochondrial HG1 and HG2; skylines’ *y* axis is the effective population size, *N*_*e*_*μ*; **B)** Timeline shows sea level variation with respect to modern times (black line) [30] and sea-surface temperature (dotted line) [31] of the last 500000 years. Yellow and light-blue bands in the lower part of the graph indicate interglacial and glacial periods, respectively, from Marine Isotope Stage (MIS) 1 to 13. Red arrows indicate events associated to COI HG1 and green to COI HG2. Green and red areas represent confidence intervals for the estimated geographic expansion date. Haplogroup divergence started over 450000 years ago during a glaciated period (MIS12). First diverged HG2 and HG3, and later diverged COI HG1, in MIS8, also associated to a cold period. The more recent geographic expansions of the two derived COI haplogroups coincides with a sea level high-stand, during MIS5. Both haplogroups expanded in the vicinity of 100000 years ago.

Demographic structure differed between data sets (Table 5). All COI, COI HG1, and COI HG2 had significant Tajima’s *D* and Fu & Li’s *F* values, which indicate a disequilibrium condition due either to the effects of natural selection or to a demographic and/or geographic expansion [27-29]. The five northern localities of COI HG1 had similar demographic signature based on Tajima’s *D* and Fu & Li’s *F* values and on their mismatch frequency distributions (Additional file 1: Figure S4). HG2 has more heterogeneous mismatch distributions per site, suggesting greater demographic independence between sites. Mismatch frequency distribution analyses of COI HG1 and HG2 had higher *P* values associated with the geographic rather than with the demographic expansion model (Additional file 1: Table S5), and thus, the geographic model was used to estimate expansion times for the five northern sites of the COI HG1 and all COI HG2 data. Based on a 3% Myr^-1^ rate of substitution for COI, the estimated expansion times derived from the mismatch distribution’s τ values were 124000 years ago for the five northern sites of HG1 and 69000 years ago for HG2 (Additional file 1: Table S5). Bayesian skyline plots displayed congruent estimates of expansion times (Figure 5a).

**Table 5:** **Estimated demographic parameters for COI haplogroups and EF1a haplotypes of *Pyura chilensis***

				**Demographic expansion**				**Geographic expansion**			
	**Site**	***D***	***F***	**SSD**	***P *** **(SSD)**	**r**	***P *** **(r)**	**SSD**	***P *** **(SSD)**	**r**	***P *** **(r)**
**All COI**	PA	**-2.09**	**-3.01**	0.006	0.20	0.031	0.15	0.006	0.20	0.031	0.45
	CP	-1.66	-2.43	0.014	0.30	0.015	0.60	0.014	0.15	0.015	0.60
	PC	**-1.96**	**-2.68**	0.012	0.25	0.013	0.75	0.012	0.10	0.013	0.95
	LH	-1.19	-2.26	0.019	0.20	0.008	0.90	0.019	0.20	0.008	0.50
	PV	-1.69	-2.51	0.017	0.20	0.013	0.55	0.017	0.25	0.013	0.75
	TL	-0.38	-1.22	0.035	0.25	0.024	0.55	0.029	0.45	0.024	1.00
	LM	0.70	0.09	0.020	0.00	0.009	0.30	0.024	0.35	0.009	0.95
	AC	-0.99	**-2.62**	0.050	0.00	0.031	0.15	0.037	0.45	0.031	0.85
	ALL	-1.73	**-3.49**	0.194	0.00	0.008	1.00	0.025	0.20	0.008	0.90
**HG1 (COI)**	PA	**-2.09**	**-3.01**	0.006	0.24	0.031	0.21	0.006	0.22	0.031	0.23
	CP	**-2.10**	**-3.40**	0.001	0.74	0.019	0.54	0.001	0.74	0.019	0.65
	PC	**-2.29**	**-3.21**	0.001	0.83	0.019	0.75	0.001	0.85	0.019	0.68
	LH	**-2.11**	**-3.21**	0.005	0.23	0.013	0.92	0.001	0.94	0.013	0.90
	PV	**-2.24**	**-3.26**	0.002	0.66	0.020	0.58	0.002	0.62	0.020	0.53
	TL	**-2.21**	**-3.19**	0.169	0.02	0.048	0.95	0.009	0.60	0.048	0.70
	LM	-1.53	-1.70	0.028	0.27	0.084	0.29	0.025	0.43	0.084	0.38
	AC	-1.18	-1.07	0.031	0.35	0.109	0.56	0.019	0.56	0.109	0.77
	ALL	**-2.56**	**-5.13**	0.0003	0.51	0.018	0.56	0.0002	0.83	0.018	0.62
**HG2 (COI)**	CP	-0.81	-0.78	0.134	0.23	0.528	0.23	0.125	0.46	0.528	0.38
	PC	**-1.20**	-0.88	0.052	0.52	0.167	0.70	0.053	0.60	0.167	0.79
	LH	-1.70	-1.99	0.007	0.71	0.042	0.70	0.007	0.80	0.042	0.77
	PV	-1.12	-1.16	0.093	0.40	0.230	0.60	0.867	0.37	0.230	0.76
	TL	-0.44	-0.68	0.014	0.78	0.046	0.87	0.019	0.70	0.046	0.90
	LM	-0.76	-0.61	0.063	0.15	0.242	0.15	0.060	0.12	0.242	0.16
	AC	-0.66	-0.84	0.037	0.14	0.088	0.25	0.043	0.13	0.088	0.42
	ALL	**-2.05**	**-4.00**	0.0055	0.64	0.018	0.88	0.0077	0.64	0.018	0.92
**HG 3 (COI)**	LM	-1.60	-1.92	0.004	0.57	0.015	0.84	0.004	0.58	0.015	0.80
	AC	-	-	-	-	-	-	-	-	-	-
	ALL	-1.53	-1.67	0.0044	0.47	0.016	0.72	0.0044	0.46	0.0156	0.77
**EF1a**	PA	0.62	0.20	0.010	0.15	0.064	0.16	0.009	0.12	0.064	0.17
	CP	0.19	0.29	0.002	0.74	0.020	0.87	0.003	0.66	0.020	0.94
	PC	0.06	-0.88	0.008	0.38	0.040	0.44	0.008	0.39	0.040	0.59
	LH	0.03	0.14	0.008	0.09	0.069	0.08	0.008	0.04	0.069	0.07
	PV	0.02	-0.28	0.013	0.28	0.059	0.34	0.011	0.41	0.059	0.49
	TL	0.24	0.03	0.005	0.27	0.045	0.25	0.005	0.31	0.045	0.31
	LM	-0.32	-0.58	0.016	0.24	0.062	0.28	0.013	0.42	0.062	0.42
	AC	-0.20	-0.98	0.005	0.20	0.034	0.43	0.004	0.30	0.034	0.47
	ALL	-0.27	-0.13	0.007	0.10	0.044	0.05	0.006	0.05	0.044	0.10

Tajima’s *D* (*D*); Fu & Li’s *F* (*F*); Significant values in bold. Sum of square deviation of the mismatch frequency distribution of the number of pairwise nucleotide differences (SSD); probability associated to SSD (*P*(SSD)); Harpending’s raggedness index (*r*); probability associated to r (*P*(*r*)). Values of SSD and r calculated for mismatch distribution expectations according to the demographic expansion model and the geographic expansion model.

## Discussion

### Isolation with divergence followed by expansion and admixture

Phylogenetic analyses of COI sequences of *P. chilensis* revealed three reciprocally monophyletic haplogroups along the Chilean coast, which are currently sympatric. The nuclear gene EF1a showed that COI haplogroups are admixed, especially the two that are geographically widespread. The divergence of the haplogroups coincides with low sea level and glaciated periods of the Pleistocene. During low sea level periods bays and shallow sections of the coast could become isolated, as peninsulas and other coastal features, and thus, could cause vicariant events. A likely scenario is that *P. chilensis* underwent isolation with divergence of three COI haplogroups due to vicariant events associated to low sea level. Divergence of HG1 and HG2 (from the basal HG3) occurred at separate times, MIS 8 and 12 of the Pleistocene, respectively. Pleistocene glaciations have had a relevant role in diversification patterns of *P. chilensis*, as well as other marine species or species complexes [9,22,23]. For example, the mud crab *Scylla paramamosain*, the sand goby species complex *Pomatoschitus minutus*, and the spinefoot fish *Siganus fuscescens,* all had population reductions associated to sea level regressions during the early Pleistocene [18,20,21].

After divergence in isolation, the two COI haplogroups of *P. chilensis* that are currently in sympatry, expanded their geographic ranges during sea level high-stands of the late Pleistocene. Tajima’s *D*, Fu & Li’s *F* values, mismatch frequency distributions, and Bayesian skyline plots, evidenced expansions of COI HG1 and COI HG2. Estimated expansions were 124000 (COI HG1) and 69000 (COI HG2) years ago, during the last interglacial and sea level high-stand (MIS 5). In this time, sea level was higher than in the present time [32-34] (Figure 5b). The LGM did not leave strong genetic signatures nor did it erase signatures left by the previous paleoclimatic events. Consistent with the inferences made for *P. chilensis*, many marine taxa had a constant population size throughout the last glaciations and LGM, with population expansions predating the LGM (e.g., [9,35-37]). Sea level increases have been suggested to trigger demographic and/or geographic expansions in many marine taxa whose expansions pre-date the LGM [18,36-38]. For example, populations of the mud crab *S. paramamosain,* and many haplogroups of goby *Sicyopterus lagocephalus,* experienced expansions during the same interglacial (MIS 5) that affected expansions of *P. chilensis*[18,37]. Water quality and shoreline characteristics change with sea level, which can lead to strong differences in habitat quality and population connectivity. Most importantly, sea levels raises would abolish vicariant barriers that may have persisted throughout low sea level glacial periods [18]. In *P. chilensis*, sea level variations would have led to the current sympatric distribution of the three COI haplogroups that diverged in allopatry.

Some of the individuals of the two widely distributed and sympatric COI haplogroups show mixed ancestry. Bayesian clustering analyses of the SNPs of the nuclear EF1a gene showed that two of the three ancestral clusters identified were admixed in some individuals, indicating that the COI haplogroups did not become reproductively isolated from the acquired divergence. The topology of the EF1a gene tree of individual sequences (Figure 1d) was also consistent with an admixture scenario; almost half of the EF1a sequences of the individuals of COI HG3 (47,37%) grouped closer to the individuals of the other COI haplogroups. In the absence of admixture, all COI HG3 individuals would have grouped separately from HG1 and HG2. There was a much higher degree of admixture between COI HG1 and COI HG2. Probably, secondary contact between COI haplogroups during interglacial times of the Late Pleistocene allowed nuclear admixture in *P. chilensis*. Further analyses with more variable nuclear markers should highlight the degree of admixture of each pair of the COI haplogroups along the Chilean coast.

EF1a did not show evidence of strong selection, and in specific aspects, EF1a showed a concordant pattern with the COI data. Mainly, both markers showed that the local population in LM was highly differentiated (discussed further on). However, phylogenetic analyses showed a mismatch between the information content of each maker. It is worth noting that discordance between markers from different genomes has been frequently reported for other taxa, becoming a common and expected pattern in animals (e.g., [39,40]). Both incomplete linage sorting and nuclear admixture-introgression can shape these seemingly contrasting phylogenetic patterns [39]. On the one hand, the isolation period that led to the COI divergence may have not been long enough to erase shared ancestral polymorphisms in the more conserved EF1a gene. The mitochondrial genome has a faster lineage sorting because it has one quarter of the effective population size of the nuclear genome, and incomplete lineage sorting is usually invoked when explaining significant differences in the patterns of differentiation of mitochondrial and nuclear sequences [39], as the one observed between COI and EF1a of *P. chilensis*. On the other hand, detected phylogeographic patterns and nuclear admixture suggest that the discordant patterns of differentiation between mitochondrial DNA and the EF1a gene are also a consequence of the nuclear admixture that occurred after secondary contact of the COI lineages, when they became sympatric.

#### Some genetic structure, but less than expected

The degree of diversity encountered in the haplotype data was high even for ascidians e.g., [41,42], which are known to bear high genetic diversity presumably due to their large effective population size [43] and fast mutational rate [23]. In spite of the degree of variation, and the short dispersal larval stage, in general, *P. chilensis* had low levels of differentiation and no isolation by distance was detected for any data sets. Genetic differentiation and isolation by distance are expected when larval dispersal limits connectivity in species with wide geographic distributions. The short-lived larvae of *P. chilensis* should lead to greater differentiation with increasing geographic distance if larvae are the only means of connectivity. Although lack of isolation by distance pattern does not directly imply connectivity, the detected diversity and structure (generalized lack of strong genetic differentiation, absence of phylogeographic break at 30°S, degree of admixture, and lack of isolation by distance), considered in conjunction with the life history of the species, shows that local populations of *P. chilensis* differ from the pattern expected for low dispersers in the HCS along the coast of Chile (e.g., [14-16]). Since *P. chilensis* is a hull biofouling species, the generalized low genetic structure, detection of admixture between two lineages, and lack of isolation by distance pattern, could be the consequence of effective gene flow mediated by maritime transport. Commonly, species that disperse as biofouling show patterns of genetic structure that are dissociated with geographic distance. For example, the worldwide COI genetic structure of the tunicate *Styela plicata*, a biofouling species, did not follow a pattern of isolation by distance, and there was non-significant structure between ocean basins [41]. Many ascidians have spread by biofouling [41,42,44-48] and as *P. chilensis*, they tend to have high genetic diversity (e.g. [32,44,49,50]).

EF1a SNP data and haplotype data of COI and EF1a revealed that LM was unique with respect to the other analyzed local populations. LM showed high genetic diversity (harboring the three COI haplogroups) and significant genetic differentiation. Additionally, LM was the only site out of Hardy-Weinberg equilibrium (EF1a SNP data), which could be due to strong evolutionary forces or to a Wahlund effect. Each divergent group within LM was in HWE when analyzed separately, supporting a scenario of a spatial Wahlund effect in LM, i.e. population substructure and cryptic diversity. In addition to the genetic evidence of a Wahlund effect, cross-fertilization is the predominant reproductive strategy of *P. chilensis*[2], reducing chances of inbreeding. Heterozygote deficit linked to Wahlund effects have been reported several times for ascidians [40,41,44,45,49], now summing *P. chilensis* to the number of known ascidians that harbor cryptic diversity, at least in LM. Another possible explanation for a strong apparent Wahlund effect is that LM has been subject to restocking from source populations with differing genetic structure, and that restocking has been successful. However there are no records associated to formal restocking plans in the area to validate this possibility.

The uniqueness of LM, with high diversity and highly differentiated with both genes, could be due to lower maritime transport. Ship routes along the coast of Chile connect most localities analyzed, except LM that is located in a protected area distant from main shipping routes [30] (Additional file 1: Figure S5). The distance of LM to the main routes of transportation could explain the lower connectivity and higher differentiation of this locality. The possible effects of biofouling on the genetic structure of *P. chilensis* have to be further investigated with an appropriate sampling scheme and highly variable markers.

For management purposes it is worth noting that the southern area of distribution of *P. chilensis* harbored greater diversity, for the overall COI data and for the EF1a data. Thus, the two molecular markers with different modes of inheritance detected that the southern area of the geographical distribution of *P. chilensis*, particularly LM, harbored an overall greater diversity and was highly differentiated from the rest. Using allozymes, Astorga and Ortiz [3] found that *P. chilensis* from Puerto Montt (PM), the southernmost of the three locations analyzed (at 41°28′S), was the most differentiated. PM is at a latitude similar to AC from this study, however it is located on the eastern side of Chiloe, in a more protected area. The most protected site we analyzed was LM. Posibly *P. chilensis* from PM could be genetically similar to those from LM, considering their more sheltered habitat. Given the high diversity and differentiation, the area around PM, described in Astorga and Ortiz [3], and LM, studied herein, should be genetically characterized at a mesoscale to define population limits and estimate connectivity. The southern area should be considered as a strategic area in management plans. If restocking has to be programmed, plans should avoid affecting the genetic diversity in the vicinity of LM (and PM until further studied), or use it as a source area. Ideally, movement of individuals between sites should be restricted to pairs of sites that are genetically/demographically undifferentiated and that have similar relative frequencies of COI haplogroups.

## Conclusions

The use of mitochondrial and nuclear markers allowed detection of divergent mitochondrial haplogroups and nuclear admixture revealing no reproductive isolation between COI lineages. Both the geographic isolation that led to the divergence of COI haplogroups and the geographic range shifts that led to current sympatry and admixture of COI haplogroups seem to have been forced by Pleistocene’s glacial and interglacial cycles.

Hull fouling cannot be discarded as a means of dispersal for *P. chilensis* given that there is a general homogeneity. The only site that had significant mitochondrial and nuclear differentiation, Los Molinos (LM), was the one located the furthest from main maritime routes. The distance of LM to the main routes of transportation could explain the lower connectivity and higher differentiation of this locality. If restocking of *P. chilensis* has to be programmed, plans should avoid affecting the high and unique genetic diversity in the southern area of its distribution, particularly in Los Molinos (LM).

## Methods

### Sampling, DNA extraction, amplification and sequencing

Samples were obtained via scuba diving from eight localities in the coast of Chile (Table 1). For each specimen approximately 10 mm^3^ of tunic tissue was preserved in absolute ethanol. DNA was extracted from 25 mg of tissue using the standard phenol-chloroform procedure [51].

Partial sequences of the mitochondrial gene Cytochrome Oxidase I (COI), were obtained using the primers HCO and LCO [52]. The gene COI is often used in phylogeographic [53] and Barcoding (e.g. [54]) projects given its high interspecies divergence and relatively low intra-species divergence. As a nuclear counterpart, we developed primers and obtained sequences of the Elongation Factor 1 alpha (EF1a) gene. This gene has shown to have a relatively fast evolutionary rate in invertebrates, and has provided informative characters for phylogeographic reconstructions (e.g. [4,9,55-57]). Contigs from 454 transcriptomic data of *Pyura chilensis* (Haye & Gallardo-Escárate, unpublished data) were used for GO analysis using Blast2Go [58]. The contig matching the EF1a gene was used to design primers for the EF1a gene for *Pyura chilensis* using Geneious R6 [59]. The primers EF1aPchF (TTGCGATCTTTTCCGCGATTGCT) and EF1aPchR (TGGGCTATATACGCAACGCTACGA) produced successful amplifications and were used to obtain partial EF1a sequences for individuals of each COI haplogroup. Primer pairs for both genes were used in PCR performed in a final volume of 10 μl with 1X PCR buffer, 1.3 Mm of MgCl_2_, 0.6 μM of each primer, 0.2 mM of each dNTP, 0.03 U μL-1 of *Taq* polymerase [Fermentas], 1.5 mg mL^-1^ Bovine Serum Albumin, and 20 ng of DNA template. Cycling conditions consisted of an initial soak of 10 min at 94°C followed by 35 cycles, each of 1 min at 94°C, 1 min at 40°C and 2 min at 72°C, and a final extension of 72°C for 13 minutes. Sequencing of purified amplicons was performed with an ABI 3730XL capillary automated DNA sequencer [Applied Biosystems].

Sequences were aligned using Geneious R6 aligner and were visually inspected and corrected, if necessary, using amino acidic sequences (translation) as a guide. All polymorphic sites were carefully inspected to call for the right base. EF1a sequences were inspected for heterozygotes carefully and the proper IUPAC one letter code was assigned to indicate the pair of nucleotides of each site that was variable within an individual. All sequences obtained were deposited in the GenBank nucleotide database [COI GenBank accessions: KC918366 - KC918536; EF1a GenBank accessions: KC936798 - KC936876].

Complete sequences, as well as each codon position, were analyzed for mutation saturation using DAMBE 5.3.16 [60]. Deviations from neutrality were analyzed from synonymous and nonsynonymous mutations with z-test codon-based test of neutrality (difference between nonsynonymous and synonymous substitution rates) calculated with the Nei-Gojoboti method [61], and Fisher’s exact test of neutrality based on sequences also with the Nei-Gojoboti method [62,63]; both performed in MEGA 5.2 [64].

The haplotypic phases of EF1a sequence data were determined using PHASE 2.1 [65] implemented in the software DnaSP 5.10 [66]. Five independent runs were performed with 10000 iterations, a thinning interval value of 10 and a burn-in period of 10000. The best results were selected based on the overall goodness of fit. Haplotypes that had a posterior probability of resolution ≥ 90% were chosen for further analyzes. Additionally, genotypic data of EF1a SNP loci with over 5% of polymorphism (based on minor allele frequency) was generated by visual inspection of chromatograms.

### Phylogenetic analyses

Sequences of the COI gene were analyzed all together as well as by COI haplogroup. For phylogenetic analyses of EF1a sequences, and to represent the variability within individuals, the nucleotide sequences analyzed included heterozygotes, denoted by the corresponding IUPAC letter code. Analyses were performed with the PAUP* 4.0b10 [67] and MrBayes 2.6.0 [68] plugins for Geneious R6. Trees were estimated using Bayesian inference (BI), Maximum likelihood (ML), and Maximum Parsimony (MP) optimization criteria. The best-fit molecular evolution model for each dataset (all COI sequences, each COI haplogroup and EF1a sequence data) was chosen with Akaike Information Criterion implemented in jModelTest 0.1.1 [69]. Chosen models were applied to BI and ML analyses. Support values for nodes of ML and MP were obtained from Bootstrap analyses with 10000 replicates. Bayesian posterior probability values were obtained from two independent runs of 1000000 iterations that led to an average standard deviation of split frequencies between chains below 0.01. Twenty percent of the trees were discarded as burn-in.

ML, BI and MP analyses were performed on a COI data set consisting of the 100% consensus sequence of each haplogroup, and sequences of three outgroup taxa of the genus *Pyura* (GenBank accessions in Figure 1b). Unfortunately there were no EF1a sequences available to polarize the EF1a gene tree.

The time of divergence of the COI haplogroups was estimated using the average number of differences between the sequences of haplogroups obtained using DnaSP. Tunicates have a high mutation rate of mitochondrial encoding sequences when compared to other bilaterians [23], although no specific values have been published. Even though for marine invertebrates a 1.5% Myr^-1^ mutation rate is considered relatively high, a fastest 3% Myr^-1^ mutation rate is likely closer to the real mutation rate for ascidians, which has been described as more than double the “typical” marine invertebrate rate (e.g., [23,24]). Additionally, several authors have pointed that intraspecific mutation rates are much greater than substitution rates between species as mutation rates decrease over time [25,26]. However, it is not yet clear how to correctly estimate given the existing variation between taxa and genes. Under the above considerations we applied a divergence rate of 3% Myr^-1^ to COI data of *Pyura chilensis*. This rate triples the one usually used for COI of marine invertebrates (1% Myr^-1^) and doubles what is generally considered a fast rate (1.5% Myr^-1^).

### Genetic structure of EF1a SNPs

From the EF1a SNP data, exact test for Hardy-Weinberg equilibrium for each locus and each population were calculated using the Markov chain method with 5000 iterations implemented in Genepop v4.1.4 [70,71] followed by sequential Bonferroni correction [72]. Observed and expected heterozygosities per locus per population, population level *F*_*ST*_, and pairwise *F*_*ST*_ were obtained with Arlequin 3.5 [73] and inbreeding coefficients (*F*_*IS*_) with Fstat 2.9.3.2 [74].

Bayesian clustering analysis was performed in STRUCTURE 2.3.4 [75] using the linkage model of ancestry [76]. Ten independent runs for each genetic cluster (*K*) (1 to 8) were performed with 500000 MCMC iterations after a burn-in of 50000. The delta *K* method was used for the inference of the best *K*[77].

### Genetic structure of haplotypes

Genetic structure was evaluated for all the COI data, each of the COI haplogroups detected with phylogenetic analyses, and for the EF1a haplotype data. DnaSP software was used to estimate haplotype and nucleotide diversities, average number of differences between pairs of sequences and Hudson’s Snn [78]. Haplotype frequency per site was obtained from DnaSP. Arlequin was used to estimate genetic differentiation of populations with Φ_ST_ (*F*_*ST*_ analogue that accounts for haplotype frequency and divergence) with 10000 permutations. The spatial analysis of molecular variance implemented in SAMOVA 1.0 [79] was performed to estimate the number of population groups; statistical significance was evaluated with 1000 random permutations. The most likely number and composition of population groups were chosen based on the probability and F_*CT*_ values. Isolation by distance was tested with a Mantel test between genetic differentiation and geographic distance [80] using 10000 permutations in Arlequin. The correlation between linearized genetic differentiation and geographic distance was also explored.

Tajima’s *D*[27] and Fu & Li’s *F*[81] neutrality tests were used to infer demographic history of all the haplotype datasets. Significance was estimated using a beta distribution in DnaSP. A negative Tajima’s *D* value is expected if populations have experienced an expansion. The timing of demographic events was inferred from two methods: Mismatch Distribution Analyses [28] and from Bayesian Skyline plots [82]. Mismatch distributions of the frequency of nucleotidic differences between pairs of sequences, used to detect historical population expansion [29], and calculation of the Raggedness index and its associated probability, were performed in Arlequin. The position of the mean (τ) in unimodal distributions corresponds to the start of the population expansion. Timing of the expansion can be calculated with the relationship τ = 2 μ*t*, where μ is the substitution rate for the whole haplotype per generation and *t* is time in generations. Analyses of mismatch frequency distribution of pairwise differences between sequences [28,83] are very conservative because they use little information of the data set [84]. Consequently, they were only performed for haplogroups that had a signal of possible demographic expansion based on neutrality tests. From the obtained τ values, as well as the upper and lower boundary of τ, COI haplogroup expansions were estimated using mutation rates of 1.5% and 3% Myr^-1^. Past changes in population size were also evaluated with Bayesian Skyline Plots generated in BEAST 1.7.4 [82,85]. This analysis uses a coalescent approach coupled to a MCMC sampling procedure to generate a probability distribution of past population sizes. The run consisted of 200 million iterations, sampling every 1000 MCMC steps. The initial 10% was discarded as burn-in. Convergence of data and demographic plots were performed with TRACER v.1.5 [86].

## Competing interests

The authors declare that they have no competing interests.

## Authors’ contributions

PAH and NCMH collected samples in the field. PAH conceived the idea, did phylogenetic analyses, and wrote the manuscript. NCMH performed laboratory procedures and most of the data analyses. Both authors read and approved the final manuscript.

## Supplementary Material

Additional file 1 Supporting material associated to presented results, including additional figures and tables associated to COI and EF1a data analyses. Click here for file
