# Supplementary material for: Isolation with differentiation followed by expansion with admixture in the tunicate Pyura chilensis
Source: BMC Evol Biol. 2013 Nov 15;13:252. doi: 10.1186/1471-2148-13-252 (PMC3840596; doi:10.1186/1471-2148-13-252)
Supplement: Additional file 1 — Supporting material associated to presented results, including additional figures and tables associated to COI and EF1a data analyses. [file 1471-2148-13-252-S1.pdf]

Additional file 1 of 1

Supplementary Information

Figure S1 - Best-k for Bayesian clustering analysis of EF1a SNP data of *Pyura chilensis*

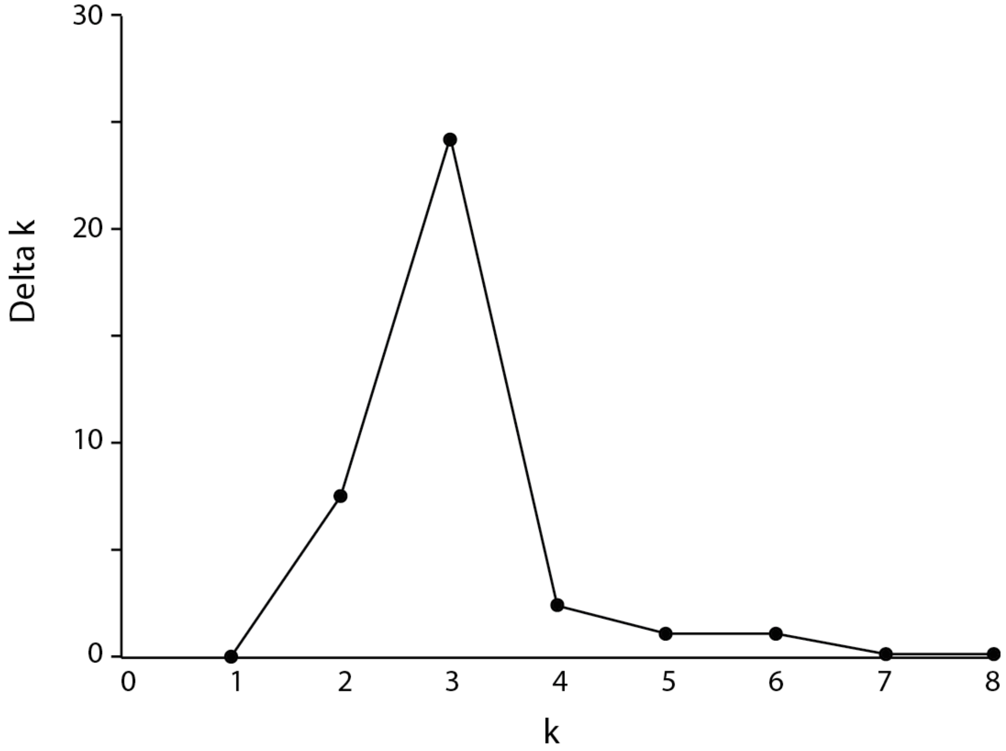

Figure S2 – Number of individuals of each COI haplogroup of *Pyura chilensis* assigned with highest probability to each of the three Bayesian k-clusters.

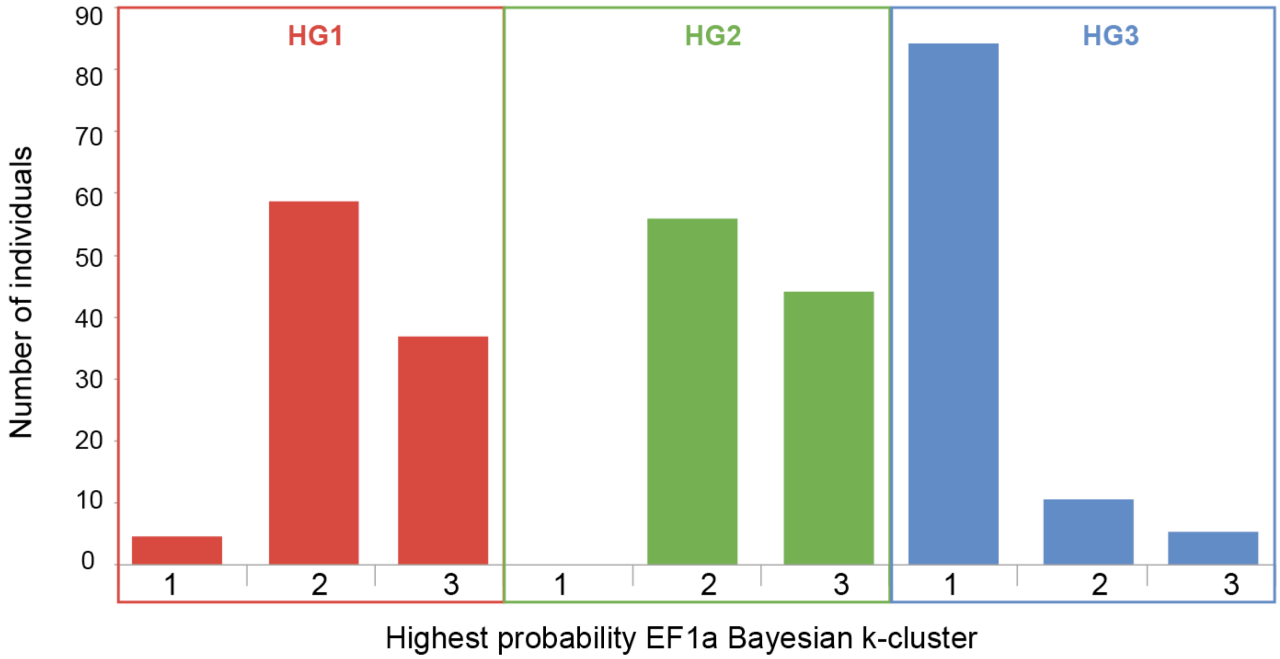

Figure S3 - Geographic distribution of the diversity of three COI haplogroups and EF1a haplotypes of *Pyura chilensis*

COI haplotypes are shown organized per haplogroup (HG1-HG3) and EF1a data corresponds to haplotypes generated with Phase. Portions in white represent haplotypes unique to one site; colors represent each haplotype and their frequency is proportional to the number of individuals that bared it.

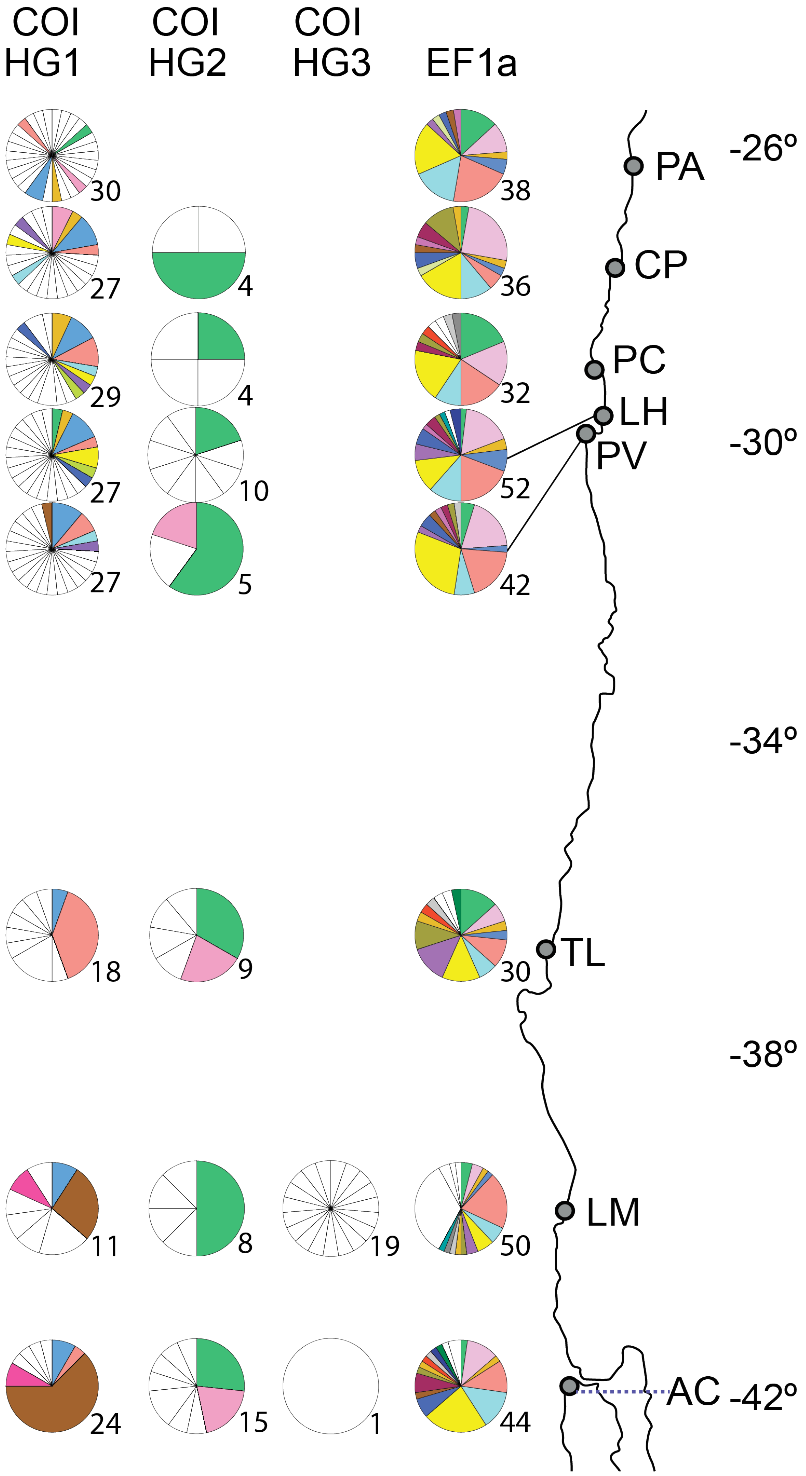

Figure S4 - Mismatch frequency distributions of COI haplogroups and EF1a haplotypes of *Pyura chilensis*

Number of observed (bars) and expected (lines) pairwise differences between sequences under a geographic expansion model.

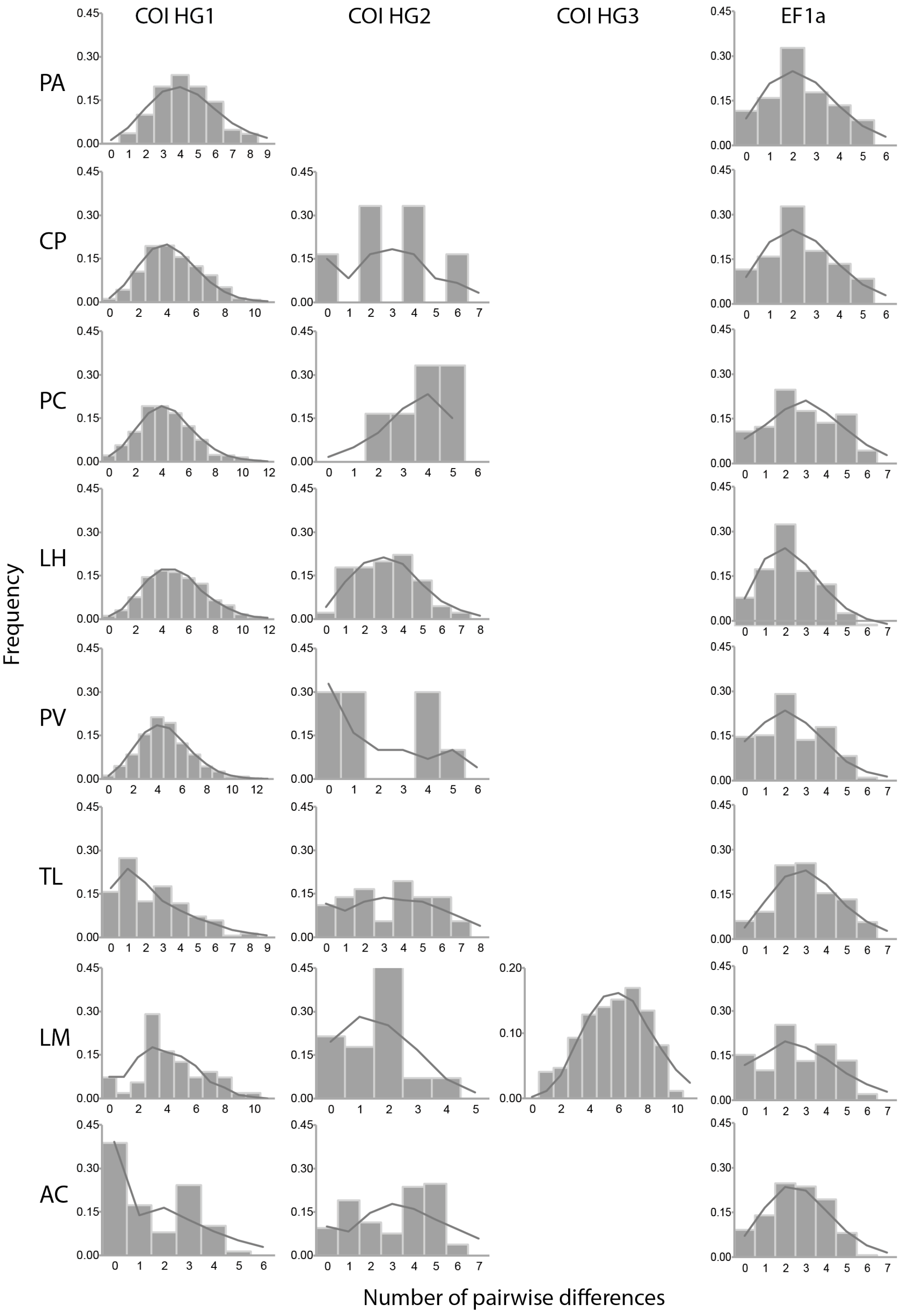

Figure S5 - Maps of marine transport and human impact on the study area

A) Marine commercial activity (shipping) map modified after the downloadable worldwide version of provided at http://www.nceas.ucsb.edu/globalmarine/impacts (Halpern et al. 2008). B) Map of the model of Halpern et al. (2008) of global map of human impact on marine ecosystems, modified in Google Earth after the open access version of the model (http://www.nceas.ucsb.edu/globalmarine/models). White dots represent sampling sites.

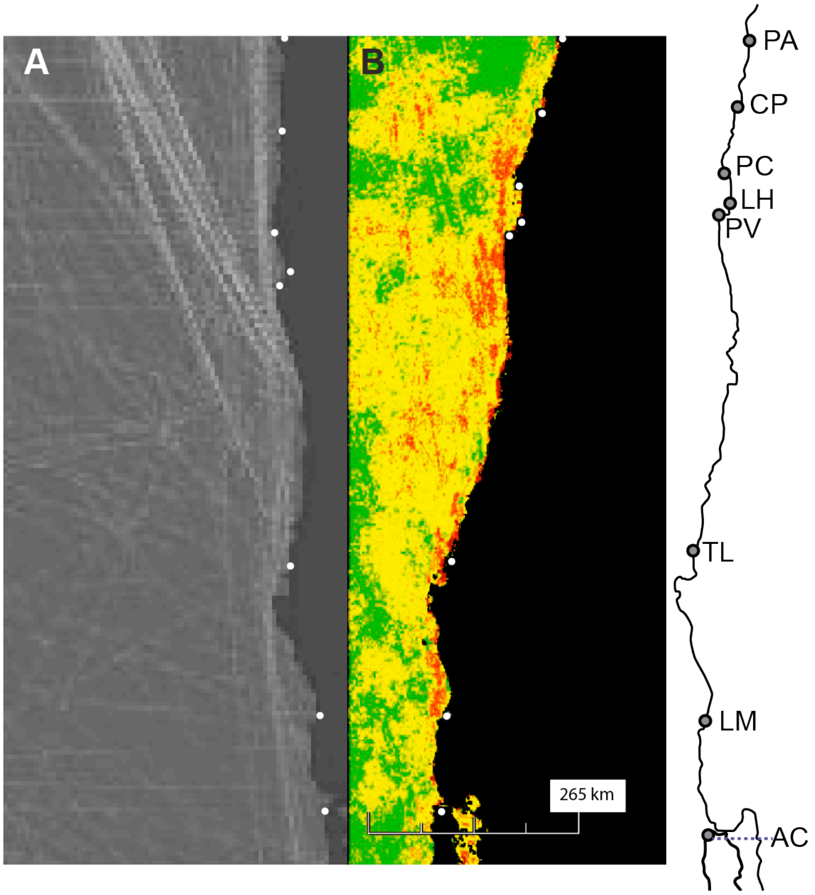

REFERENCE:

Halpern B, Walbridge S, Selkoe KA, Kappel CV, Micheli F, D'Agrosa C, Bruno JF, Casey KS, Ebert C, Fox HE, et al.: **A global map of human impact on marine ecosystems**. *Science* 2008, **319**:948–952

Table S1 - Locus by locus probability of Hardy-Weinberg equilibrium for EF1a SNPs of *Pyura chilensis*.
Significant departures after Bonferroni correction in bold ($P < 0.05$).

	PA	CP	PC	LH	PV	TL	LM	AC
EF1a52	0.898	0.914	0.635	0.831	0.810	**0.004**	0.868	0.900
EF1a58	0.440	0.586	0.464	0.526	0.733	0.790	**0.003**	0.431
EF1a113	M	0.909	0.865	0.900	M	M	**0.007**	M
EF1a114	M	M	M	M	M	M	**0.008**	M
EF1a115	0.621	0.919	0.495	**0.01**	0.398	0.854	**0.004**	0.171
EF1a136	0.323	0.231	0.115	0.552	0.619	0.863	**0.005**	0.722
EF1a169	0.873	0.509	0.890	0.677	0.785	0.855	**0.006**	0.747
EF1a184	0.560	0.571	0.256	0.903	0.396	0.197	0.319	0.950
EF1a271	0.171	0.911	0.811	**0.003**	0.724	0.341	0.191	0.790

Table S2 - Locus by locus F_{IS} inbreeding coefficients for EF1a SNPs of *Pyura chilensis*
Significant values in bold.

	PA	CP	PC	LH	PV	TL	LM	AC
EF1a52	0.000	-0.057	-0.073	-0.018	-0.021	**0.618**	-0.094	0.037
EF1a58	-0.125	-0.083	-0.128	-0.098	-0.043	-0.030	0.478	-0.130
EF1a113	NA	0.000	0.000	0.000	NA	NA	**0.805**	NA
EF1a114	NA	NA	NA	NA	NA	NA	**0.863**	NA
EF1a115	-0.071	0.034	-0.117	**0.456**	-0.147	0.000	**0.507**	-0.242
EF1a136	0.201	-0.209	0.343	0.125	-0.076	0.064	**0.721**	-0.047
EF1a169	0.000	-0.106	0.000	-0.057	-0.021	0.064	0.178	-0.040
EF1a184	-0.089	0.125	0.254	0.028	0.186	0.325	-0.154	0.019
EF1a271	-0.237	0.003	-0.023	**0.563**	-0.047	0.247	0.242	0.066
All	-0.089	-0.029	0.060	0.**233**	-0.019	0.195	0.476	-0.050

Table S3 - Pairwise multi-locus F_{ST} for EF1a SNPs of *Pyura chilensis*

Significant values in bold ($P < 0.05$).

	PA	CP	PC	LH	PV	TL	LM
**PA**							
**CP**	**0.05248**						
**PC**	-0.00014	0.01542					
**LH**	0.01595	0.01004	0.00934				
**PV**	-0.00898	0.01943	-0.01311	0.00609			
**TL**	0.02002	0.03403	-0.00049	0.03528	0.00262		
**LM**	**0.1471**	**0.15236**	**0.14322**	**0.11485**	**0.14456**	**0.14842**	
**AC**	0.02026	-0.00039	-0.00944	0.00401	0.00066	0.00584	**0.13826**

Table S4 - SAMOVA groupings for COI haplogroups and EF1a haplotypes of *Pyura chilensis*

	K	2	3	4	5	6	7
**ALL COI**	F_{CT}	0.449	**0.340**	**0.284**	**0.259**	**0.247**	**0.236**
	P	0.124	0.042	0.019	0.012	0.019	0.032
	Groups	1. LM	1. LM	1. LM	1. LM	1. LM	1. LM
		2. rest	2. PA	2. TA	2. PA	2. PA	2. PA
			3. rest	3. AC	3. TA	3. TA	3. TA
				4. rest	4. AC	4. AC	4. AC
					5. rest	5. LH	5. LH
						6. rest	6. PV
							7. CP,PC
**COI HG1**	F_{CT}	0.105	**0.078**	**0.061**	**0.049**	**0.044**	**0.043**
	P	0.122	0.036	0.018	0.016	0.005	0.028
	Groups	1. AC	1. AC	1. AC	1. AC	1. AC	1. AC
		2. rest	2. LM	2. LM	2. LM	2. LM	2. LM
			3. rest	3. TA	3. TA	3. TA	3. TA
				4. rest	4. LH	4. LH	4. LH
					5. rest	5. PA,LV	5. PA
						6. CP,PC	6. LV
							7. CP,PV
**COI HG2**	F_{CT}	0.053	**0.058**	**0.068**	**0.086**	0.084	-
	P	0.140	0.042	0.005	0.022	0.065	-
	Groups	1. LM	1. LM	1. LM	1. LM	1. LM	
		2. rest	2. PC	2. PC	2. PC	2. PC	
			3. rest	3. CP,LH	3. TA	3. TA	
				4. rest	4. AC	4. AC	
					5. rest	5. LH	
						6. PV,TA	
**ALL EF1a**	F_{CT}	0.075	**0.061**	**0.053**	**0.048**	**0.046**	**0.049**
	P	0.123	0.046	0.014	0.002	0.025	0.049
	Groups	1. LM	1. LM	1. LM	1. LM	1. LM	1. LM
		2. rest	2. PA,CP	2. CP	2. CP	2. CP	2. CP
			3. rest	3. TL	3. TL	3. TL	3. TL
				4. rest	4. LH,AC	4. LH	4. LH
					5. rest	5. AC	5. AC
						6. rest	6. PA
							6. PC,PV

Table S5 - Signals of past demographic events and estimated times of expansion of two COI haplogroups of *Pyura chilensis*

Sum of square deviation of the mismatch frequency distribution of the number of pairwise nucleotide differences (SDD); probability associated to SDD (*P*(SDD)); Harpending's raggedness index (*r*); probability associated to r (*P*(*r*)). Values of SDD and r calculated for mismatch distribution expectations according to the demographic expansion model and the geographic expansion model. Approximate coalescent time (τ); lower (τ_{lb}) and upper (τ_{ub}) boundaries calculated with the percentile method based on 1000 bootstrap replicates at alpha = 0.05; estimated time of expansion in million years calculated with a mutation rate of 1.5% Mya^{-1} ($t_{exp-1.5}$); estimated time of expansion in million years was calculated with a mutation rate of 3% Mya^{-1} (t_{exp-3}); confidence limit of expansion time, lower boundary (CL_{lb}); confidence limit of expansion time, upper boundary (CL_{ub}).

**Model**		**COI HG1 Northern sites**	**COI HG2**
**Demographic expansion**	SDD	0.00029	0.00550
	P (SDD)	0.53000	0.64000
	r	0.01669	0.01801
	P (r)	0.39000	0.88000
	τ	4.557	3.854
	τ_{lb}	3.555	0.486
	τ_{ub}	5.182	7.592
	$t_{exp-1.5}$	0.247	0.209
	$CL_{lb-1.5}$	0.193	0.026
	$CL_{ub-1.5}$	0.281	0.412
	t_{exp-3}	0.124	0.105
	CL_{lb-3}	0.096	0.013
	CL_{ub-3}	0.141	0.206
**Geographic expansion**	SDD	0.00028	0.00771
	P (SDD)	0.61000	0.64000
	r	0.01669	0.01801
	P (r)	0.42000	0.92000
	τ	4.560	2.545
	τ_{lb}	2.822	0.745
	τ_{ub}	5.358	5.484
	$t_{exp-1.5}$	0.248	0.138
	$CL_{lb-1.5}$	0.153	0.040
	$CL_{ub-1.5}$	0.291	0.298
	t_{exp-3}	0.124	0.069
	CL_{lb-3}	0.077	0.020
	CL_{ub-3}	0.145	0.149
